# Microstructural Characterization of 3D Printed Cementitious Materials

**DOI:** 10.3390/ma12182993

**Published:** 2019-09-16

**Authors:** Jolien Van Der Putten, Maxim Deprez, Veerle Cnudde, Geert De Schutter, Kim Van Tittelboom

**Affiliations:** 1 Magnel laboratory for Concrete Research, Department of Structural Engineering, Faculty of engineering and Architecture, Ghent University, Technologiepark Zwijnaarde 60, B-9052 Ghent, Belgium; 2 PProGRess–UGCT, Department of Geology, Faculty of Science, Ghent University, Krijgslaan 281, S8, B-9000 Ghent, Belgium; 3 Chairholder “Porous media imaging techniques”, Department of Earth Sciences, Faculty of Geosciences, Utrecht University, Princetonlaan 8A, 3584CD Utrecht, The Netherlands

**Keywords:** 3D printing, mechanical properties, microstructure, pore size, durability

## Abstract

Three-dimensional concrete printing (3DCP) has progressed rapidly in recent years. With the aim to realize both buildings and civil works without using any molding, not only has the need for reliable mechanical properties of printed concrete grown, but also the need for more durable and environmentally friendly materials. As a consequence of super positioning cementitious layers, voids are created which can negatively affect durability. This paper presents the results of an experimental study on the relationship between 3DCP process parameters and the formed microstructure. The effect of two different process parameters (printing speed and inter-layer time) on the microstructure was established for fresh and hardened states, and the results were correlated with mechanical performance. In the case of a higher printing speed, a lower surface roughness was created due to the higher kinetic energy of the sand particles and the higher force applied. Microstructural investigations revealed that the amount of unhydrated cement particles was higher in the case of a lower inter-layer interval (i.e., 10 min). This phenomenon could be related to the higher water demand of the printed layer in order to rebuild the early Calcium-Silicate-Hydrate (CSH) bridges and the lower amount of water available for further hydration. The number of pores and the pore distribution were also more pronounced in the case of lower time intervals. Increasing the inter-layer time interval or the printing speed both lowered the mechanical performance of the printed specimens. This study emphasizes that individual process parameters will affect not only the structural behavior of the material, but they will also affect the durability and consequently the resistance against aggressive chemical substances.

## 1. Introduction

The exploration of using additive manufacturing in the construction industry started in the mid-1990s, when Pegna [1] tried to implement this technique at the Rensselaer Polytechnic University in New York. He investigated the bonding between Portland cement and sand layers using steam to accelerate the curing process in a solid freeform fabrication. He tried to illustrate this method with simple masonry structures that could not be obtained by casting. This was a first improvement in reducing the construction time, but it was Khoshnevis [2] who developed a 3D printing system specific to construction (contour crafting) where it became possible to deposit layers of concrete-like filaments on top of each other.

Within this newly developed 3D printing (3DP) technique, there is a significant interdependency between material, process and final product. This interdependency is even more pronounced when using a cementitious material for two reasons. First, the slow setting reaction of the printed concrete results in a strong interaction with the applied print parameters, such as pump pressure and printing speed. Secondly, concrete in itself does not have a single fixed composition, but can have a wide range of compositions that may be more or less suitable in relation to the printing process and the required properties of the final product [3,4,5]. Through the years, many researchers have investigated different mix compositions in order to obtain a printable concrete, having the advantages of self-compacting concrete and sprayed concrete at the same time. However, before any revolution becomes new practice, there is a period of study and optimization, and most researchers have been faced at a certain point with the conflicting requirements the material has to fulfill. First, there is the workability and printability that demand a good flow in the printer tubes and nozzle. More specifically, the printing material may not settle too fast in the reservoir and blocking or segregation in the tubing must be prevented. Secondly, buildability requires stability of the printed layers: being viscous enough, and not setting too fast to benefit the bonding surface and not too slow to obtain enough strength to sustain its own weight and the weight of superposed layers without relevant deformations. Currently, innovations in printing process and material development are evolving rapidly, but apart from the additional requirements set by the new technology, the cementitious material and the printed specimens themselves face problems inherent to their mix design, independent of the procedure.

As a consequence of the lack of molding, shrinkage and more specifically (plastic) drying shrinkage will become more important and pronounced when comparing 3D printing with traditionally cast concrete. Plastic shrinkage starts immediately after mixing and is related to the transport of moisture to the environment. Due to the absence of molding, both the sides and the interface between the printed layers are insufficiently protected, resulting in evaporation of water at the concrete surfaces. This loss needs to be counteracted by the water present inside the cementitious mixture. However, if the water content is not sufficient and if the water does not cover all the surface particles, capillary pressure will develop due to the formation of water menisci in the pores. Consequently, this pressure will give rise to capillary tensile stresses that may lead to cracking [6,7]. At early stages, when the concrete is still a formable mass, these cracks are shallow and have an irregular shape. Considering the fact that in the case of 3D printed elements both the sides and the interface are exposed to the atmosphere, this increases crack occurrence and ingress paths for chemical substances. Another characteristic of this technique is the layered structure of the end product in which voids can form in between the different filaments. These voids are another preferential ingress path for aggressive substances which will again not only weaken the structural behavior of the printed element but will also decrease its durability.

In addition to the occurrence of cracks and the inclusion of voids through the layered construction process, the pores created during hydration of the cement paste will also play a key aspect when studying the durability of printed elements. As reported by Mehta and Monteiro, the pore structure of cementitious materials can be classified into gel pores, capillary pores and air voids. These pores have different dimensions, are formed during various hydration stages, and will also affect the fresh and hardened properties of the material. For example, the smallest pores (gel pores) will affect shrinkage and creep, while the bigger pores (capillary pores) will affect more the compressive strength and permeability of the cementitious material [8]. This pore classification is not new, but the formation and the distribution of these pores will be different in the case of 3DP due to the predefined time gap. From the moment fresh material is deposited, the hydration and structural build-up starts, and both the interface and surroundings of the printed layer will become drier over time due to the exothermal hydration reaction and the absence molding. Another consequence is the occurrence of a moisture exchange phenomenon. When the first deposited layer becomes drier over time, it absorbs more water from the freshly deposited layer on top of it. This extra water can influence the hydration of the bottom layer and, simultaneously, some air present inside the bottom layer escapes. This air stays entrapped at the interface, again affecting the mechanical properties and durability of the printed element.

For 3D printed concrete and related construction processes, the effect of the manufacturing process on the hardened properties has already been investigated by various authors, but the effect on the microstructure and durability has not yet been investigated in depth and many parameters in this research field are still unknown. The current study aims to comprehend the correlation between the process parameters and the developed microstructure of printed elements, both in fresh and hardened states. In particular, the effect of two printing process parameters (inter-layer time interval and printing speed) on the porosity and microstructure was established. In the fresh state, a series of tests was conducted to demonstrate extrudability, buildability and structural build-up capacity of the chosen mix composition. Results in the hardened state were based on mercury intrusion porosimetry (MIP) and air void measurements to characterize the pore size and pore size distribution. X-ray micro-computed tomography (µCT) scanning was performed to visualize the pore distribution through the printed element. The microstructure was correlated with the mechanical performance of printed elements to obtain a better understanding of the structural behavior and durability.

The effect of the applied print process (e.g., the effect of different printing speeds) was evaluated based on measuring the surface roughness of the specimen using the automated laser measuring (ALM) technique [9].

## 2. Materials

The 3D printable cementitious material contains ordinary Portland cement (OPC) CEM I 52.5 N (Holcim, Belgium), normalized siliceous sand (0/2), water and a polycarboxylate superplasticizer (SP) (Glenium 51, conc. 35%, BASF, Germany) to increase the flowability of the mix. The mixture composition is based on the research of Khalil [10] and can be found in Table 1. The chemical and mineralogical composition of the cement is given in Table 2. The mineralogical composition is calculated based on Bogue equations.

## 3. Methods

### 3.1. Mortar Preparation

First, dry cement and sand were mixed for 30 s at 140 rotations per minute (rpm) with a planetary mortar mixer (Macben, Belgium). Then, water and superplasticizer were added and mixed for 30 s without changing the mixing speed. To ensure a homogeneous mixture, the speed was increased to 285 rpm for the next 30 s. Afterwards, the edges of the bowl were scraped for 30 s and the mixture was allowed to rest for 60 s. The final step was mixing the mortar for 60 s at high speed. This preparation method was valid for all the tests except for the calorimetric tests. The preparation method used for calorimetry is mentioned in Section 4.1.3 (2) ‘Calorimetry’.

### 3.2. Printing Procedure

**Figure 1:**
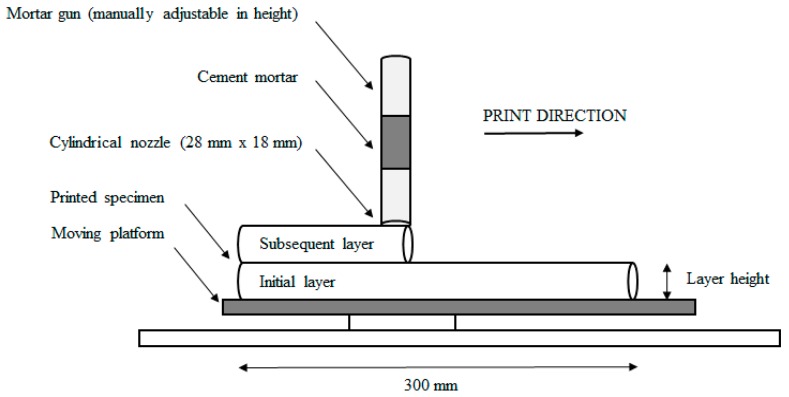
Schematic illustration of the extrusion-based 3D printing process.

Printed specimens were prepared by using a custom-made apparatus (Figure 1), equipped with a Quickpoint mortar gun with a Black & Decker DR250 3/8” Drill, able to simulate the extrusion-based 3D printing process on a smaller scale. The developed system is equipped with an elliptical nozzle (28 mm × 18 mm) capable of printing layers with a maximum length of 300 mm on top of each other at different speeds. For the purpose of this study, two linear printing speeds (e.g., 1.7 cm/s and 3.0 cm/s) were selected. The layer height is manually adjustable, and to ensure the same print quality in both cases, the height of the layers was fixed at 15 mm and 20 mm for a low and high printing speed, respectively. Consequently, a different printing speed and layer height also result in a different flow rate. In the case of the low printing speed, the flow rate was equal to 0.028 m^3^/h, and for the higher printing speed the flow rate amounted to 0.065 m^3^/h.

Sample preparation consisted of filling the printing apparatus and extruding the material through the nozzle with a constant speed. A single base layer, with a length of approximately 300 mm was extruded for each specimen. After a predetermined time interval (0, 10, 30 or 60 min), another layer was deposited on top of the previous one. In case of a 0 min time gap, two layers were printed from the same mortar batch. However, for any time gap, fresh mortar was extruded on top of the first layer in order to ensure the same behavior of the mixture. After changing the vertical position (Z-direction) of the nozzle manually, the printing/deposition of the second layer started at the same horizontal position (X-direction) to create a similar time gap in between the printed layers at every position. After printing, the specimens were stored for 28 days in a standardized environment (20 ± 3 °C, 60% RH).

## 4. Characterization Methods

### 4.1. Fresh State Characterization

Three-dimensional printing applications require favorable characteristics of the cementitious material in the fresh state and these properties should be maintained during the complete 3D printing process. The choice of an optimal mix is therefore the foundation of the success of further applications, and a deeper and more detailed understanding is necessary due to the highly demanding requirements of the material [11].

#### 4.1.1. Extrudability

A first critical parameter is the extrudability of the mortar. This parameter describes the ability of the mixture to be extruded through the nozzle and deposited as an even and continuous filament with almost no deformations. As no standard characterization methods are available, the extrudability was evaluated based on the layer deformation immediately after extrusion. Based on Kazemian [12], deformations of the cross-sectional width within a range of 10% were accepted. It should be noted that these dimensional limitations are set for fresh mortars and are measured manually for every specimen at three different positions directly after printing.

#### 4.1.2. Buildability

Buildability, or early age stiffness, is another critical parameter and refers to the ability of the material to retain its shape under self-weight and the weight of superposed layers. Proper buildability was obtained when five layers of the cementitious material could be printed on top of each other [10,11,12,13,14]. Buildability also depends on the workability and mix proportions and, in particular, the variation in workability over time (also called open time). In this research, buildability is quantitatively assessed by measuring the start and end of setting using an automated Vicat apparatus (EN 196-3). For this test method, the mortar is placed in a 40 mm high Vicat mold with an internal diameter equal to 75 mm. A needle with a 1 mm^2^ cross section penetrates the mortar sample every 15 min, measuring the penetration depth and correlated resistance. The initial and final setting times are specified based on the measured penetration depth, more specifically as the time at which the distance between the needle and the base plate of the mold is respectively equal to (6 ± 3) mm and 0.5 mm.

Besides this destructive test, it is also possible to measure initial and final setting on a non-destructive and more precise manner using a Freshcon apparatus [15,16,17]. This ultrasonic transmission system is able to determine the wave velocity, wave energy and the frequency content when ultrasonic pulses go from the one to the other transducer positioned on opposite sides of the fresh mortar specimen [15]. A mortar container (35 cm^3^) consisting of two poly(methyl methacrylate) plates, tied together by four screws and spacers was used. The mold was a U-shaped rubber foam element with high damping properties. A contact agent (multi-purpose silicon grease) was used between the sensor and the protective wear cap to prevent the creation of air bubbles. The same grease was used between the protective wear cap and the mortar. During the measurements, the specimen was covered with plastic foil to avoid water evaporation and shrinkage. Every 5 min, an electric pulse was sent by the data-acquisition card from the computer through the amplifier (450 V for mortar specimens) to the piezoelectric broadband transmitter (central frequency 0.5 MHz) generating an ultrasonic wave. The start and end of setting can be determined by comparing the acquired velocity and energy graphs. For the purpose of this research, the start of setting was determined as the inflection point in the velocity graph and the time at which the energy ratio E/E_ref_ of 0.01 was reached [16]. In this formula, E is the wave energy through the mortar and E_ref_ is the energy through the container filled with water [18]. The end of setting was determined as the time at which E/E_ref_ became 0.07 [16]. These measurements can be correlated to the TAM AIR measurements (see Section 4.1.3 (2)) as they also permit following the cement hydration and monitoring the development of the microstructure at an early age [18].

#### 4.1.3. Structural Build-Up Mechanism

The structural build-up of mortars is governed by complex and coupled physical and chemical phenomena: flocculation and hydration. In case of printed mortars, the space between the sand particles is saturated with matrix material and, consequently, the development of the yield stress is governed by the behavior of the cement paste. For cement pastes, attractive interactions between cement grains leading to flocculation and the growth of hydration products at the contact points between the cement grains are the main mechanisms leading to yield stresses [5]. These two processes can be measured respectively by performing a uniaxial unconfined compression test (UUCT) or by calorimetric measurements.

(1) Uniaxial unconfined compression test

The primary factors affecting the yield stress due to flocculation in a dense suspension are the colloidal interactions and Calcium-Silicate-Hydrate (CSH) nucleation at the contact points between the cement grains [19]. It is a reversible physical phenomenon that takes place in the induction period directly after water addition and gives the mortar this thixotropic behavior, which will increase the strength and stiffness of the material. As thixotropy can affect the inter-layer bond strength due to the formation of cold joints, it is necessary to measure the structuration at different mortar ages. During the experiments, the tested mortar ages are kept equal as the applied time gaps in order to find a correlation between both. This means that UUCT is performed at mortar ages of t = 0, 10, 30, and 60 min. Here t = 0 min is the earliest time possible taking into account demolding, placing of the specimens in the test setup and starting the test, which takes approximately 5 min. UCCT were performed on cylindrical samples (Ø = 25 mm, h = 20 mm), produced by filling a plastic mold after mixing the cementitious material, which was carefully demolded just before testing. The test specimens were loaded in a Walter + Bai DB 250/15 machine under displacement control. Bearing in mind the application, the displacement-controlled tests were performed at a rate of 48 mm/min, which mimics the loading rate during printing and allows the test to be performed fast enough to neglect effects of thixotropic build-up. In total, at least three specimens were tested for each mortar age.

(2) Calorimetry

Hydration is an irreversible chemical phenomenon in which the formed hydrates bond the cement grains together and create the supporting structure of the mixture. The primary factor affecting the yield stress due to hydration is the formation rate of the hydration products [5]. Observations of the heat of hydration can therefore reveal information about the chemical reactions taking place in the cement [20]. In this research, dry components (OPC + Sand), previously stored at 20 °C, water and SP were mixed manually for 15 s outside the calorimeter and were afterwards immediately placed inside the measuring device. The mortar reactivity was determined through isothermal calorimetry measurements at 20 °C using a TAM AIR isothermal heat conduction calorimeter (TA instruments). Values of the hydration heat were registered every 30 s for 7 days.

### 4.2. Surface Roughness

At the age of 28 days, the surface roughness of the printed specimens was determined for both printing speeds by measuring the height of the surface peaks and valleys with a high precision laser beam (resolution = 5 µm), mounted on an in-house developed ALM table [9]. The ALM device was equipped with two stepping motors, controlling the motion in both the X- and Y-directions. The profile measurements were used to calculate the center-line roughness (R_a_ [no unit]) of the printed layers with an accuracy of 7 µm. This value is determined with an average line, drawn through the measured profile. R_a_ is then calculated as the sum of the surface areas a_i_ [no unit] between the profile and the center-line over a selected reference length (Equation (1)).

(1) $$R_{a}\left[-\right]=\frac{1}{n}\overset{n}{\underset{i=1}{\sum }}\left|a_{i}\right|$$

**Figure 2:**
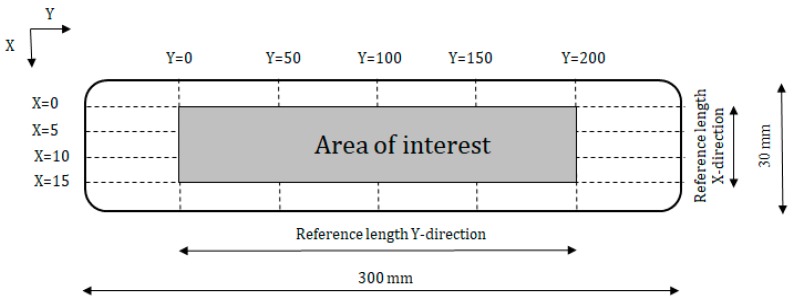
Schematic representation of automated laser measurement (ALM) technique (dimensions in mm).

For the purpose of this research, the reference length is equal to 200 mm in the Y-direction and 15 mm in the X-direction. This reference length was selected to include important roughness features but exclude errors of form. As can be seen in Figure 2, roughness measurements were performed every 5 and 50 mm in the X- and Y-directions, respectively.

### 4.3. Surface Moisture

The evolution of the surface moisture content of a printed filament was observed for 1 h by gently pressing blotting paper strips on top of it at predefined time steps (0, 10, 30 and 60 min after printing) and measuring their mass change. Aurora ref.10 blotting paper, with an areal density of 125 g/m^2^, was cut into three equal strips (7.5 cm × 2.5 cm) and placed on the surface of the printed specimen.

The mass of absorbed fluid per unit area k [g/cm^2^] of one paper strip is obtained by following the subsequent steps:

- The paper strip is weighed (m_dry_ [g]) with a precision of 0.001 g, placed on the printed surface at a predefined time and pressed onto it by means of a plastic weight for 60 s. The weight exerts a uniform pressure of 77.5 ± 0.1 Pa;
- The paper strip is weighed to obtain the mass after possible absorption (m_sat_ [g]);
- The mass of the absorbed fluid m_abs_ [g] at a certain time is obtained by Equation (2):
  (2) $$m_{\mathrm{abs}}\left[g\right]=m_{\mathrm{sat}}-m_{\mathrm{dry}}$$
- The exact surface of the paper strip (A_strip_ [cm^2^]) is obtained by reverse calculation knowing the areal paper density and the initial dry weight of the strip;
- The mass of absorbed surface fluid k can be calculated based on Equation (3).
  (3) $$\kappa \;\left[g/\mathrm{cm}{}^{2}\right]=\frac{m_{\mathrm{abs}}}{A_{\mathrm{strip}}}$$

The maximal area mass of absorbed water k_max_ (g/cm^2^) is obtained by spraying water on the paper strips until complete saturation. It is used as a reference value which cannot be exceeded by those resulting from the above testing procedure.

### 4.4. Mechanical Performance

#### 4.4.1. Compressive Strength

**Figure 3:**
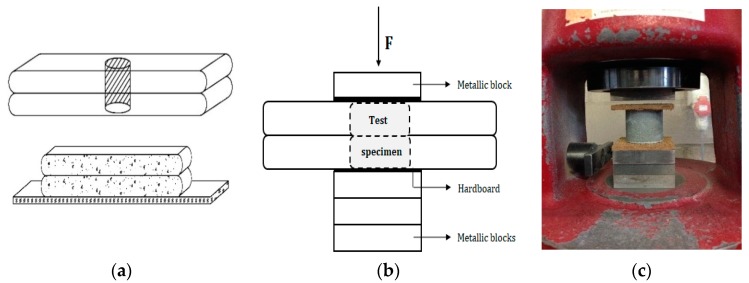
Compressive strength test setup: (**a**) cylindrical test samples); (**b**) schematic representation; (**c**) complete test setup.

The mechanical properties were evaluated based on compressive strength and inter-layer bonding strength after storage for 28 days. The compressive strength f_c_ [N/mm^2^] was measured for at least three cylindrical specimens (Ø = 25 mm, h = 20 mm, Figure 3a), drilled out of a printed element and loaded perpendicular to their print direction using the test machine Walter + Bai DB 250/15 under load control at a rate of 100 N/s. As previous research [21,22] showed the highest compressive strength in the perpendicular direction, further investigation of the anisotropic behaviour is not included in this research. To obtain representative results, the evenness of the top and bottom surface plays an important role, and for that reason both surfaces were smoothened before testing. Due to the fact that the samples were very small, hardboard is used during this test to reduce irregularities (Figure 3).

#### 4.4.2. Inter-Layer Bonding Strength

**Figure 4:**
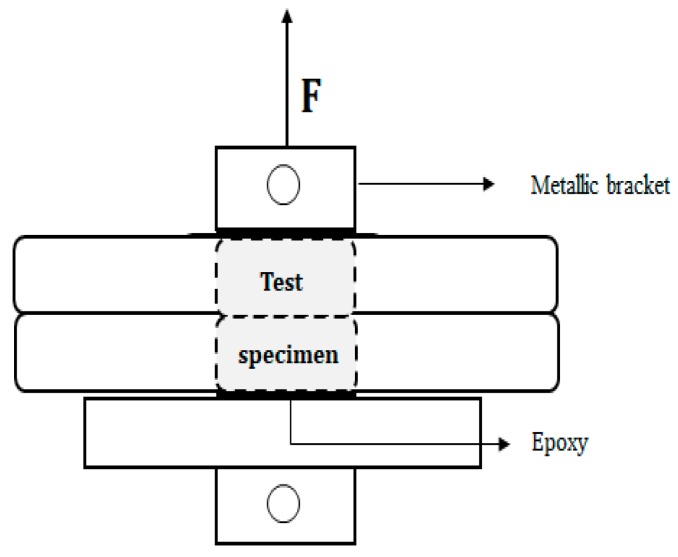
Schematic illustration of the inter-layer bond test setup.

A schematic representation of the inter-layer bond strength test setup is shown in Figure 4. For every time gap, at least three cylindrical specimens (Ø = 25 mm, h = 20 mm, Figure 3a) were drilled out of a printed element and tested with a Macben Proceq pull-off machine under displacement control at a rate of 50 N/s. On the top and bottom surface, two metallic brackets were glued with epoxy and special attention was taken to align the specimens in order to avoid any eccentricity and associated scatter in the results.

### 4.5. Microstructure

#### 4.5.1. Optical Microscopy

**Figure 5:**
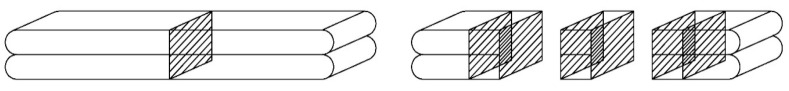
Thin section specimen preparation.

Correlations between the surface roughness and the applied printing speed were based on optical microscopy. Therefore, thin sections (40 mm × 25 mm × 30 µm, Figure 5) were prepared in order to study the sand particle distribution through a printed sample.

The thin sections were impregnated with fluorescent epoxy (Stuers NV.), scanned afterwards using a Leica DMPL microscope with DFC 295 camera and analyzed with ImageJ. Different regions with a cumulative height of 0.5 µm, starting from the surface, were selected and the amount of sand particles was calculated within the different areas. For both printing speeds, one thin section was analyzed. Within each sample, a total of eight areas was used to calculate the changing sand volume.

#### 4.5.2. Pore Size Characterization

The description of the pore structure is a key aspect when studying the durability of cementitious materials. Based on their size, the pores can be classified into gel pores, capillary pores and air voids. Unfortunately, one single test method to characterize the entire pore structure is at this moment still a utopia and is non-existent. Therefore, different test methods (both in 2D and 3D) are applied to characterize and visualize the pore structure through a printed specimen. As the dimensions of the gel pores are too small to provide relevant information, they are not taken into account in this research.

(1) Mercury Intrusion Porosimetry

To study the capillary porosity (in the range of 0.1 nm < d < 100 µm), mercury intrusion porosimetry (MIP) was performed (Pascal 140 and 440 series, Thermo Fisher Scientific Inc.). As a specific pressure corresponds to an aperture of a pore, and the amount of mercury intrusion approximates to the pore’s volume, the number and size of pores could be determined in 3D. MIP does not directly measure the number of macro pores, but macro pores show up in the total porosity of the measured sample.

**Figure 6:**
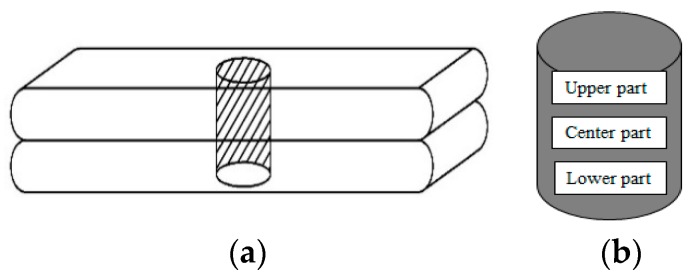
Cylindrical test samples (**a**) and representation of the different zones (**b**) measured through a printed specimen during mercury intrusion porosimetry (MIP) analysis.

During MIP measurements, mercury is forced into a printed specimen of approximately 1.5 g with a random shape. These small specimens are obtained by sawing a drilled cylindrical specimen (Ø = 14 mm, h = 20 mm, Figure 6a) into smaller pieces. These pieces are obtained from the upper, center and lower part (Figure 6b) of a drilled specimen to characterize the effect of the applied time gap on the capillary pores.

The pressure needed to force the mercury into a cylindrical pore is Equation (4):

(4) P=−4×γ×cos(θ)/d

where:
PPressure[N.m^−2^]ϒSurface energy of Hg (=0.483 N.m^−1^)[N.m^−1^]θContact angle (=140°)[°]dNominal diameter of the pore[mm]

To minimize microstructural damage during pre-conditioning, samples were freeze-dried for 1 week at the age of 28 days, and during the experiments the maximum pressure was limited to 200 MPa to avoid crack formation. For every time gap, one specimen was analyzed.

(2) Air Void Analysis

**Figure 7:**
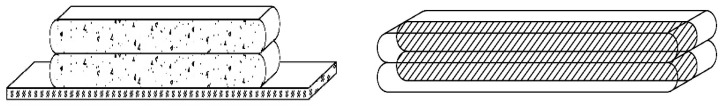
Specimen sampling for air void characterization.

The macro pores (air voids) with a minimum size of 1 µm were measured using a RapidAir 457 Device. This device allows air void analysis in 2D with a resolution of 1 to 2.5 µm in order to calculate the air content of the cementitious material, printed with different time gaps, and its distribution in the hardened state according to the EN-480 Standard. The measuring system consists of a computerized control unit (PC), a 20.1 LCD color monitor, a digital color camera and a microscopic objective. The analysis is performed on rectangular specimens with an approximated area of 15 cm^2^, cut perpendicular to the longitudinal axis (Figure 7) at the age of 28 days. For every printing speed, at least two rectangular specimens were analyzed.

Afterwards, the surface of the samples was polished and treated with black ink and barium sulphate (BaSO_4_) powder to increase the contrast between the air voids and the cementitious matrix. During analysis, the mortar specimen was moved automatically in the X-direction. In order to create a complete and relevant 2D image of the surface, one does not consider the outermost 10 pixels of the image and provides a 20-pixel overlap of the images. The thresholding level for the different images is equal to 200. The actual measurement consists of measuring the cord lengths of the cavities on the image along different lines with a total width equal to one pixel. The number of lines depends on the situation. According to EN-480, the minimum chord length in the case of a maximum aggregate size of 25 mm is equal to 2413 mm. For the purpose of this research, the number of traverse lines measured to obtain this minimal chord length was kept equal to seven for all the different samples. After scanning the complete surface, the RapidAir 457 was provided with analysis software that calculates the air content A ([vol%], Equation (5)), the specific surface area and the spacing factor. For this calculation, the paste content V_paste_ ([vol%], Equation (6)) has to be entered and, for this mix design, was equal to 47.5% [8,18,23]. To avoid irregularities in the images that would be regarded as cavities, the analysis only takes into account cord lengths bigger than 3 pixels and, consequently, voids smaller than 8 µm were not taken into account.

(5) $$A=\frac{T_{a}}{T_{\mathrm{tot}}}\left[\mathrm{vol}\%\right]$$

(6) $$V_{\mathrm{paste}}=\frac{V_{\mathrm{cement}}+V_{\mathrm{water}}}{V_{\mathrm{total}}}\times 10{}^{2}$$

where:
AAir content [%]T_a_Number of voids intersected by the traverse lines [m]T_tot_Total chord length[m]V_paste_Paste content[vol%]V_cement_Cement volume (depending on the mix design)[m^3^]V_water_Water volume (depending on the mix design)[m^3^]V_total_Total volume of the mortar mixture[m^3^]

(3) µCT-Scanning

X-ray micro-computed tomography (µCT) was performed at the Ghent University Centre for Tomography (UGCT) using HECTOR [24]. This custom-made laboratory X-ray µCT scanner comprises a micro focus directional target X-ray source, which can reach up to 240 keV and 280 W (X-ray WorX XWT 240-SE) and a large flat-panel detector (40 × 40 cm^2^; PerkinElmer 1620 CN3 CS). For these measurements, one cylindrical specimen (Ø = 14 mm, h = 20 mm) for each time gap was drilled out of a printed element and positioned between an X-ray source and an X-ray detector. An aluminum filter (1 mm) was used to filter out the low-energy X-rays from the emitted energy spectrum before passing the sample. The X-ray tube was set at a voltage of 200 keV and a power of 10 W. The obtained voxel size for the cylindrical cores was 7.98 µm. For all the samples, a total of 2400 projections over 360° with an exposure time of 1 s were made. To capture the total height of each core, three separate scans were performed along one sample and combined afterwards into one single volume with Aquila (Tescan XRE) software. The raw projections were reconstructed into 3D volumes using Octopus Reconstruction (Tescan XRE) [25]. Image analysis was done with Avizo software from ThermoFisher Scientific.

(4) Scanning Electron Microscopy (SEM)

**Figure 8:**
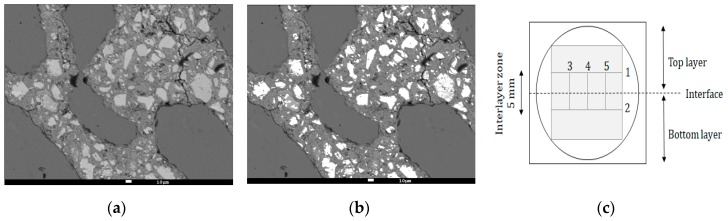
(**a**) Original BSE-SEM image; (**b**) BSE image with selected unhydrated cement (indicated in white); (**c**) schematic representation of a sample used for SEM-BSE measurements and analyses. The black and grey lines indicate, respectively, the sample border and the lines along which measurements were performed.

The formed hydration products and the hydration degree of the different layers was examined by means of a JEOL JSM-5600 SEM equipped with a BSE detector operation at an acceleration voltage of 20 kV and a magnification of 300. Small cylinders (Ø = 25 mm, h = 20 mm), drilled out of a printed specimen sawn in the longitudinal direction (Figure 8) were impregnated with a low-viscosity epoxy resin under vacuum and subsequently cured for 24 h at 35 ± 1 °C. Afterwards, the specimens were polished stepwise with SiC abrasive papers (different grindings) and coated with a carbon coating (approximately 20 nm).

After preparation, different lines on the specimen were selected for analysis (Figure 8c). For the top and bottom layer part, 20 images were taken along two parallel lines (respectively, line 1 and 2, Figure 8c) and the mean value of these results represent the calculated phases. The phases formed within the inter-layer zone, which has a defined total width of 5 mm, were calculated along three parallel lines (line 3–5, Figure 8c). For every line, a total of 10 images were analyzed every 0.5 mm. For every position within this inter-layer zone, the calculated phases are the mean value of three analyses.

Calculation of the hydration products was based on grey level histograms obtained with ImageJ. The different phases were determined as the grey level is dependent on the atomic number of the phase. All images were taken with the same brightness and contrast and based on the obtained grey levels (black 0–255 white), and three regions could be clearly distinguished: pores from 0–80, C–S–H (calcium-silicate-hydrate) and CH (calcium hydroxide) products from 101–175 and UH (unhydrated cement) from 176–255. The boundaries were determined as the distinct changes in the grey-level histogram (Figure 8).

## 5. Results and Discussion

### 5.1. Fresh State Characterization

**Figure 9:**
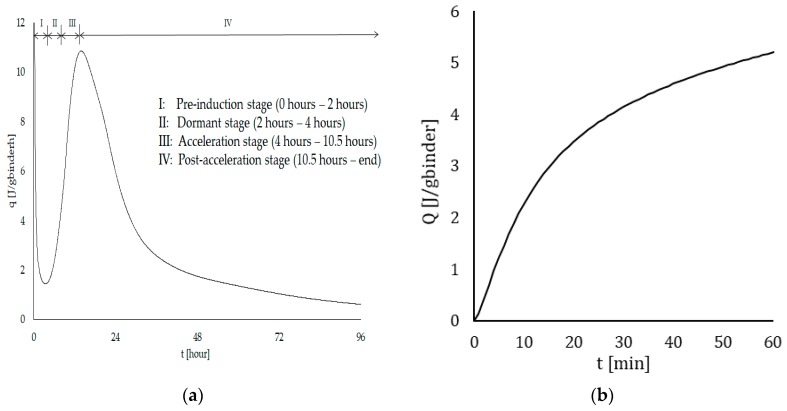
(**a**) Rate of heat evolution during the hydration of the 3D printed cementitious material; (**b**) cumulative heat release during the first 60 min after the addition of water.

Figure 9a shows the heat evolution during hydration of the cementitious material with an indication of the different stages. As this research used a normal mortar composition, the occurrence and duration of these hydration stages is very common. The first stage (pre-induction stage), characterized by the formation of ettringite and CSH can be related to the open time of the printed material. The longer this period lasts, the longer the mixture can be used for printing. This stage is followed by a period of low reactivity (dormant period). The end of this period is related to the initial setting time of the cementitious material [20] and can be derived based on ultrasonic wave velocity measurements. Correlation between the results of these two tests are confirmed within this research; more specifically the initial setting time, based on both Vicat and FreshCon measurements, is equal to 210 min (3.5 h). After the dormant stage, the hydration reaction restarts, indicating the beginning of the acceleration period, which is characterized by an onset of massive precipitation of CSH, leading to the setting and hardening of the cement. The morphology of these hydration products is generally described as prisms or long needles, ensuring the connection between the different cement particles and the build-up of the basic skeleton of the cementitious material [26]. The final setting occurs before the cement paste shows the maximum rate of heat development. Again, this is confirmed by both Vicat and FreshCon measurements, where the final setting is noticed around 410 min (6.75 h).

Figure 9b shows the cumulative heat release during the first 60 min after the addition of water and indicates that after one hour (i.e., the highest time gap applied during these experiments) the cumulative heat release is equal to 5.2 J/g_binder_, resulting in a temperature increase of approximately 0.5 °C. 

Considering the fact that these calorimetric measurements are executed in a closed system under normalized circumstances, one can rule out that the change in moisture content of the printed surface (see Section 5.2.2), which is directly and completely exposed to the environment, is affected by the heat release during hydration of the cementitious material.

**Figure 10:**
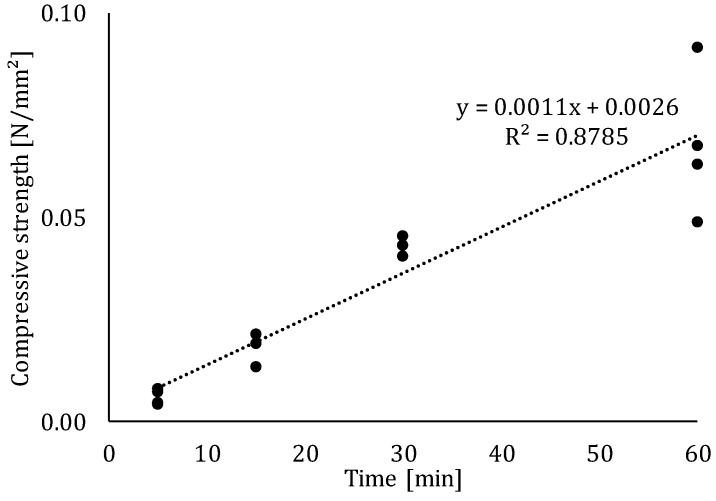
Compressive strength development of the cementitious material in the fresh state, measured at different time intervals.

Immediately after the addition of water, hydration begins followed by the formation of primary hydration products (i.e., ettringite and CSH). These products start the development of the inner structure, which will provide a certain strength to the printed material. As can be seen in Figure 10, the compressive strength at the earliest testing time is equal to 0.006 N/mm^2^, indicating that there is almost no structure build-up at that time. After 60 min, the strength increases to an average of 0.062 N/mm^2^ and a linear fit can be found between the measurements at predefined time gaps. The obtained results are comparable with those earlier reported for early age compressive strength tests [19,27].

The strength development and rate will not only affect the structural behavior at early stages but will also have an influence on the microstructure of the printed element. In the scope of this research, three different time gaps were applied (0, 10, 30 and 60 min). As stated before, in case of a 0- or 10-min time gap, the base layer is incapable of providing the required strength and will encounter the highest deformation compared to larger time gaps. In case of higher interval times, the material will sustain in a more adequate way the additional load of the second layer.

### 5.2. Surface Characterization

#### 5.2.1. Surface Roughness

Based on Table 3, one can conclude that the applied printing speed affects the surface roughness of the element in a significant way and, more specifically, printing at a lower speed introduces a higher roughness. This phenomenon will affect the mechanical properties, as discussed in a later paragraph. In case of a lower printing speed, the roughness is most pronounced in the print direction (X-direction), and for higher printing speeds the surface roughness is comparable in both directions. This difference will affect the anisotropic behavior of the printed elements but this phenomenon is not incorporated in this study.

The higher surface roughness in case of a lower printing speed can be explained based on two different phenomena: shear stress and kinetic energy. In case of a cementitious material, the material can be described as a Bingham fluid. Based on Equation (7), this type of fluid shows a linear relationship between the shear stress τ_xy_ and shear rate ϒ, and there will be no flow until a critical stress level (i.e., yield stress τ_xy_) is reached.

(7) $$\tau_{\mathrm{xy}}=\tau_{0}+\dot{\gamma }+\mu_{p}=\tau_{0}+\frac{{\mathrm{dv}}_{\mathrm{xy}}}{\mathrm{dx}}+\mu_{p}$$

where:
$\tau_{\mathrm{xy}}$Shear stress[Pa]$\tau_{0}$Yield stress[Pa]$\dot{\gamma }$Shear rate[m/s]$\mu_{p}$Plastic viscosity-v_xy_Velocity(m/s)

The yield stress and plastic viscosity are characteristics describing the material’s behavior, and can be derived based on rheological measurements. For the aim of this research, the material is kept the same and will generate the same yield stress and plastic viscosity during printing. Based on Equation (7), one can conclude that when applying a higher velocity when printing the material, a higher shear stress will be exerted on the particles of the cementitious composition, resulting in a smoother surface.

Another phenomenon that can explain the higher surface roughness for lower printing speeds is the kinetic energy, E_kin_, which is directly proportional to the mass m (kg) of a certain particle and squared proportional to its moving velocity v (m/s). Consequently, printing at different speeds will increase the kinetic energy of the particles and this effect will be more pronounced in case of sand due to the higher mass of the particles. The higher energy will force the sand particles deeper into the bulk of the printed specimen, resulting in a top layer with a smaller number of sand particles, which will create a smoother surface.

**Figure 11:**
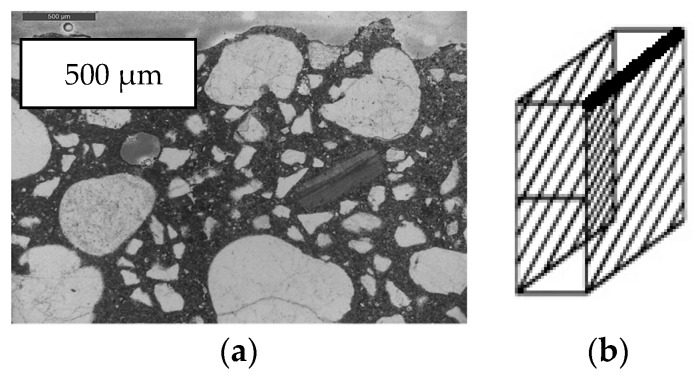
Thin section photograph (**a**) and the corresponding height within a printed specimen (black bar (**b**)).

**Figure 12:**
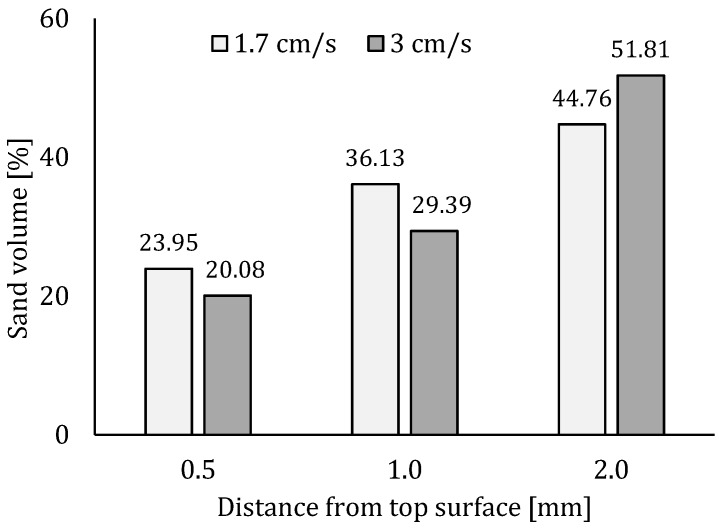
Cumulative sand volume [%] within the cement matrix in the first 2 mm of a printed sample in the case of different printing speeds.

Thin section analysis proves the latter theory. Based on a visual inspection of the thin sections, no clear distinction could be made (Figure 11a,b). However, ImageJ analysis showed that the distribution of sand particles differs spatially for both printing speeds. In case of a printing speed equal to 1.7 cm/s, the area of sand particles in the first millimeter is larger compared to the thin section created from a sample printed at higher velocity. However, beneath 2 mm, this area exceeds that obtained for the low printing speed. Based on Figure 12, one can conclude that within the second millimeter under the printed surface, the volume of sand increases with a ratio equal to 1.76, while in the case of a low printing speed this is only 1.24. This can be related to the formation of a so-called lubrication layer during pumping, which has a typical thickness of around 2 mm and where the inner bulk material contains a relatively greater number of larger particles.

#### 5.2.2. Moisture Content

**Figure 13:**
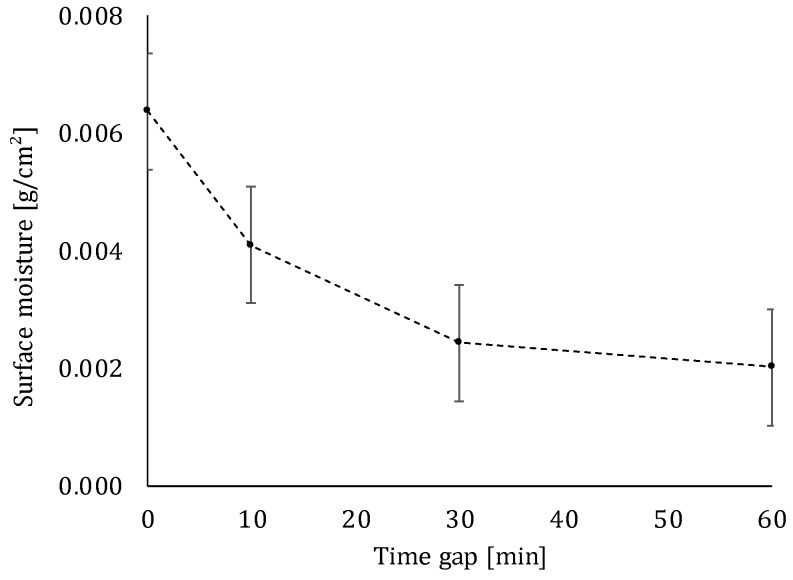
Moisture content of a printed element measured at different time gaps (error bars represent standard deviation).

After extrusion, the cementitious material is completely exposed to the environment. This means that during the proposed delay time, surface moisture evaporates and the printed layers become drier over time (Figure 13). The highest evaporation of water is observed during the first 10 min after printing (Δ = 0.00229 g/cm^2^). Afterwards, the difference in moisture content is not significant anymore. As discussed in Section 5.3, this decrease in moisture content during the first 10 min will induce the highest decrement in inter-layer bonding strength between two super-positioned layers. In case of 3D printing, the reader should keep in mind that water will evaporate from the sides, as well as from the top surface of the specimen, due to the lack of molding.

### 5.3. Mechanical Performance 

**Figure 14:**
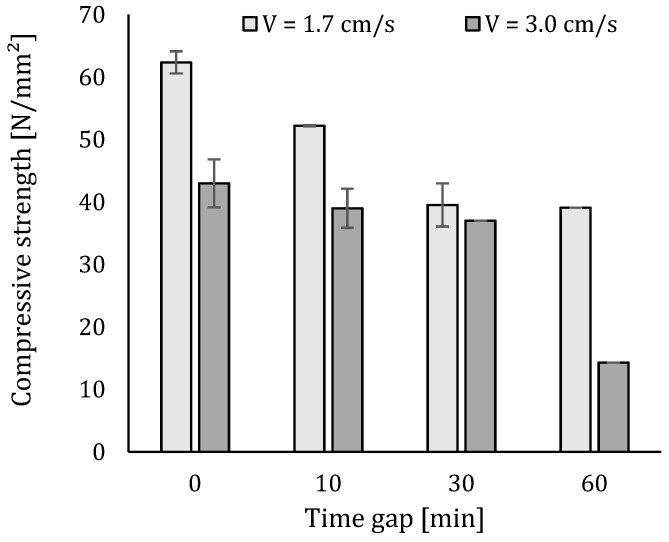
Compressive strength of 3D printed samples (error bars represent standard deviation).

**Figure 15:**
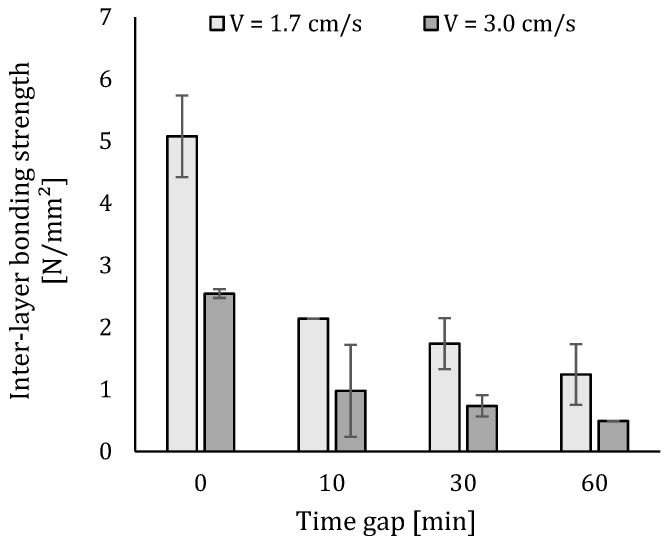
Inter-layer bonding strength of 3D printed samples (error bars represent standard deviation).

The average results of the compressive and inter-layer bonding strength are plotted in Figure 14 and Figure 15. Clearly, the strength reduces as the interval time increases. The reduction in strength for increasing inter-layer interval times is in accordance with findings by other researchers [21,28,29]. However, a quantitative comparison of these studies indicates that the inter-layer interval time cannot be considered as an independent value, but should be considered in relation to the material and other process parameters adopted in each study.

The mechanical performance of specimens fabricated with a higher speed is generally lower compared to those created with a lower printing speed. The latter can be attributed to the larger number of voids included in the printed specimen (see Section 5.4), while in the case of the inter-layer bonding strength, this decrease is caused by decreased surface roughness. The lower surface roughness will deteriorate the bonding between two super-positioned layers and consequently decrease the inter-layer bonding strength in a significant way. For that reason, only specimens manufactured with a velocity equal to 1.7 cm/s are taken into account for further investigations of the microstructure.

Comparing the inter-layer bonding and compressive strength, one can conclude that the latter is less affected by changing print parameters. The compressive strength only shows a maximum strength loss equal to 31% (strength loss between a time gap equal to 0 and 60 min), while in the case of the inter-layer bonding strength, a maximum loss between the before-mentioned time intervals of 75% is observed. The decreasing trend in compressive strength can be attributed to the higher volume of voids formed within specimens for higher time intervals, which results in a general weakening of the printed specimen. In case of the inter-layer bonding strength, the decreasing mechanical performance can be attributed to the decreasing moisture content of the surface. When the layer becomes drier, it absorbs more water from the freshly deposited top layer and simultaneously, some air inside the bottom layer escapes. This air stays entrapped at the interface and causes a poor bonding between the different layers. This phenomenon is confirmed by the fact that the highest strength loss occurs within a time gap of 0 to 10 min and, based on Figure 13, the highest evaporation of water also occurs during the first 10 min after printing.

### 5.4. Microstructure 

**Figure 16:**
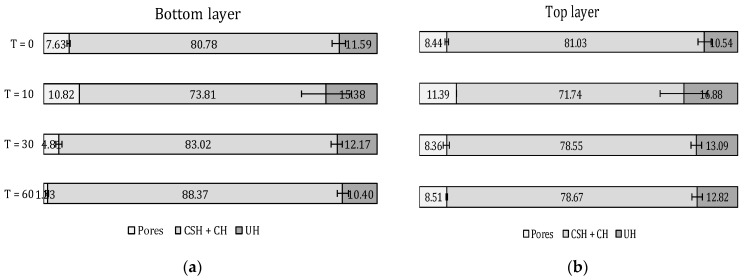
Analysis of the BSE-SEM micrographs as a quantification of the inner microstructure and present phases (capillary pores, Calcium-Silicate-Hydrate + Calcium Hydroxide (CSH + CH) and unhydrated (UH) cement; error bars represent standard deviation) at (**a**) bottom layer and (**b**) top layer of a printed specimen.

The results of the microstructural analysis based on BSE-SEM measurements on a printed specimen are depicted in Figure 16 and show a quantitative comparison of the present phases at the bottom and top layer. Focusing on the amount of unhydrated cement, one can observe that in the case of a 0-min time gap, the amount of unhydrated cement particles is comparable, both for top and bottom layers. This phenomenon confirms the fact that, as two super-positioned layers with the same age and moisture content are printed on top of each other, the moisture exchange at the interface is negligible. Increasing the inter-layer interval time induces a higher amount of UH in the top layer compared to the bottom layer. The significance of this difference between the obtained results is controlled based on one-way ANOVA tests. The higher amount of UH indicates a lower amount of water available for the hydration of cement and confirms the moisture exchange phenomenon that occurs when the layers become drier over time. The effect of the water evaporation at the sides of the printed element is not taken into account in this research.

**Figure 17:**
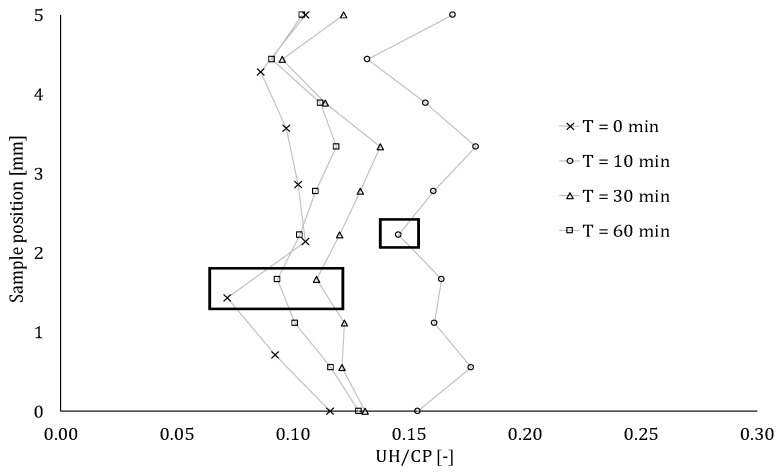
Number of UH cement particles over the total cross-section (CP) within an inter-layer zone of 5 mm. The exact position of the inter-layer for a printing speed of 1.7 cm/s, observed during BSE-SEM analysis, is indicated with rectangular areas.

The number of unhydrated cement particles at different positions in the inter-layer zone is depicted in Figure 17 for all the studied samples. Even though the standard deviation with this test is high, some qualitative conclusions may be drawn. The exact position of the inter-layer, observed during BSE-SEM analysis, is indicated by means of rectangles. In general, in case of a 0-min time gap, the number of UH cement particles is the lowest. Both layers have the same moisture content and that water evaporation through the inter-layer is blocked by printing the second layer with a 0-min time gap. Consequently, the general amount of water available for hydration is higher. This correlates with the results obtained for a 10-min inter-layer time interval. The moisture content of these elements shows the largest decrease, resulting in a higher volume of evaporated water and, consequently, UH particles. 

Focusing on the exact position of the interface, the concentration of UH decreases. This indicates that at the interface, the base layer will extract water from the super-positioned layer and this effect is more pronounced in the case of lower interval times. This phenomenon can also be explained based on the hydration products formed at that stage. In an early hydration state, the formed products are mainly CSH and ettringite, creating needles on top of the cement particles in order to create structuration bridges at a later stage. These bridges become stronger over time, increasing the mechanical performance of the element (Figure 10), after a 10-min period. Immediately after printing, these bridges are very weak and can be partly broken due to the addition of the second layer. The water exchange between those layers at an early age will therefore serve as extra water for rebuilding these structuration bridges, resulting in a higher and more pronounced effect of the hydration degree at the interface. Three millimeters above the inter-layer zone, another zone with fewer UH particles is seen. This can be due to the shear action of applying a second layer on top of the base layer, resulting in a small amount of extra water that can be used for hydration. Again, as the standard deviation is noteworthy, only a qualitative trend can be seen. Both shear and water migration cause the material to form in a different way. This was clearly noticed from the µCT results, showing a different microstructural behavior. However, given the above-mentioned assumptions, more specific calculations and analysis of the different amount of hydration products based on XRD measurements are necessary and will be the scope of further research.

In addition to the UH cement particles, BSE-SEM analysis also allows determination of the capillary pores formed during the hydration process (Figure 16). The highest volume of capillary pores is observed for a 10-min time gap. Plotting and comparing these results with the pores measured based on MIP experiments and CT scanning, the same conclusion can be made. These observations align with the assumption that lowering the moisture content of the surface will increase the moisture exchange between the layers, creating a higher capillary pore volume. As mentioned in the introduction, these capillary pores are responsible for the strength development of the cementitious material. Consequently, a higher capillary pore volume will negatively affect the mechanical behavior of the material by means of a lower strength. This phenomenon explains the decreasing compressive strength when increasing the applied inter-layer interval time (Figure 14). After a critical time gap of 10 min, the capillary porosity of the elements decreases and a comparable amount can be found in the case of 30- and 60-min time gaps.

**Figure 18:**
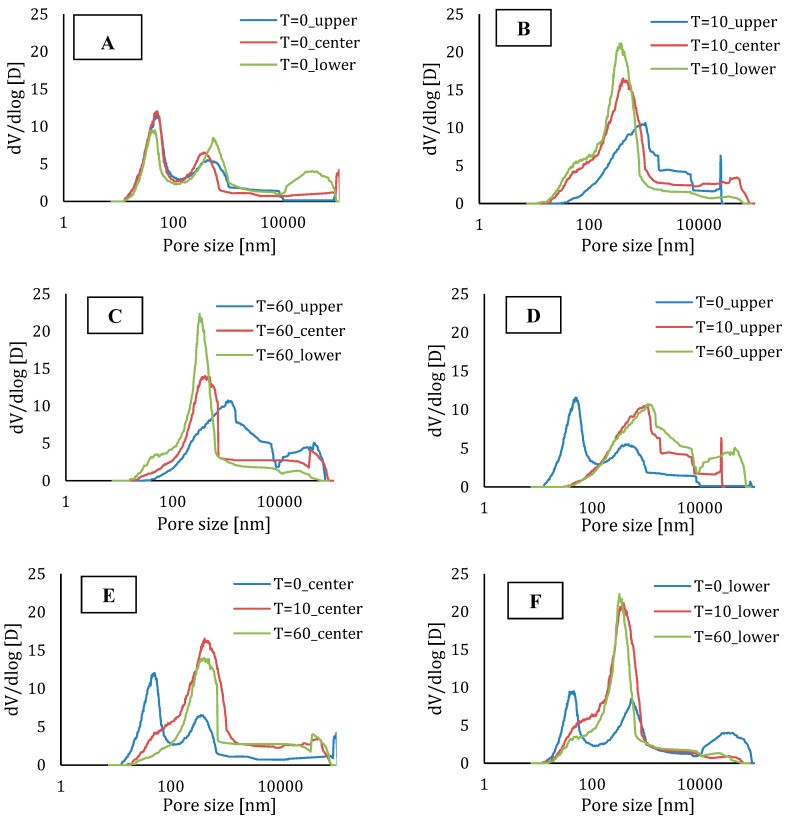
Pore size distribution of elements with different time gaps and low printing speed: Comparison between different areas in a specimen printed with (**A**) 0 min time gap; (**B**) 10 min time gap; (**C**) 60 min time gap. Comparison between (**D**) the upper part; (**E**) the center part; (**F**) the lower part of a specimen printed with different time intervals.

In addition to the above-mentioned effect of the capillary pores on the mechanical or microstructural behaviors, the pore-size distribution also plays an important role. The results of the MIP tests are shown in Figure 18. Based on Figure 18A, it becomes clear that, comparing different parts of a printed element with a time gap equal to zero, the pore-size distribution is comparable for every region in the printed specimen.

**Figure 19:**
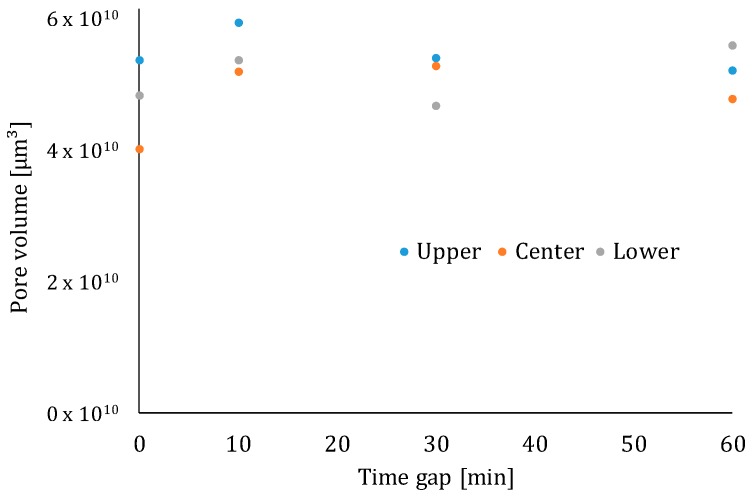
Calculated pore volume based on µCT-scanning.

For elements with a higher inter-layer time interval, the number of pores with a smaller diameter is greater in the center and lower part of the specimen (Figure 18B,C) and, in both cases, the upper part of the specimen shows more larger pores. These results are comparable with the results obtained by µCT (Figure 19). Moreover, Figure 18D–F shows a comparison between the different parts of the elements produced with different time gaps. Comparing the upper part, one can only see a shift towards larger pores when increasing the deposition time between the layers and the total porosity remains more or less the same. In the center and lower region of the specimen, one can observe both phenomena (i.e., a shift towards bigger pores and an increased porosity).

**Figure 20:**
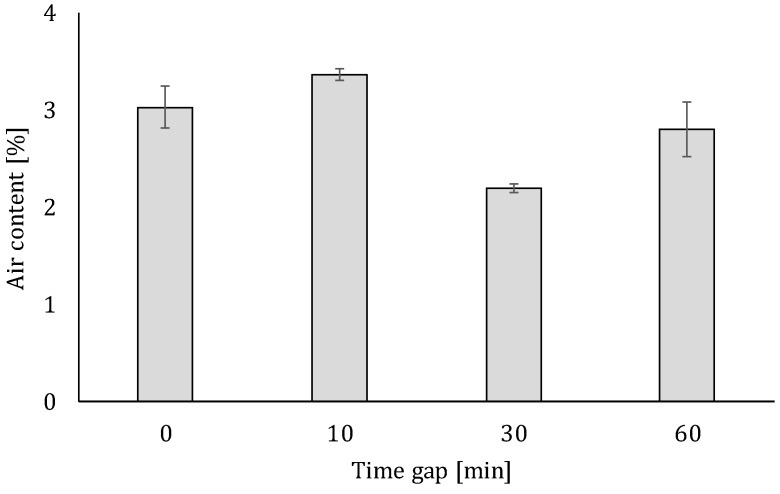
Average air content (calculated based on Equation (5)) of the hardened mortar mixtures fabricated with different inter-layer interval times (error bars represent standard deviation).

The air content of the hardened mortar mixtures is shown in Figure 20. Based on this, one can conclude that in addition to the highest capillary porosity, specimens with a time gap equal to 10 min also contain the highest amount of air. The creation of these voids, combined with the highest air content, results in the highest decrease in structural behavior.

**Figure 21:**
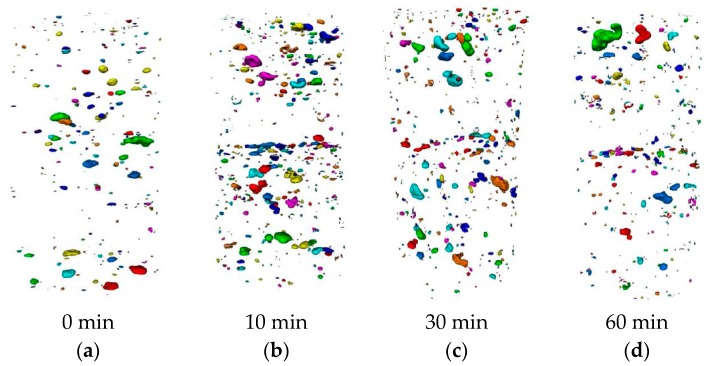
Three-dimensional rendering of air voids with a flatness between 0.1 and 0.45 obtained with Octopus reconstruction software for specimens printed with different time gaps: (**a**) 0 min; (**b**) 10 min; (**c**) 30 min; (**d**) 60 min.

µCT-scanning is also an important tool to visualize the pores in three dimensions. Figure 21 comprises 3D renderings of µCT data. Here, all voids with a flatness between 0.10 and 0.45 are displayed (0 is totally flat, 1 is round). Based on these figures, one can conclude that the morphology of the voids changes in the transition zone and that the inter-layer of the printed specimen contains more flat and elongated pores due to the moisture exchange between the different layers and the lack of structural stability in the case of a 10-min time gap. This phenomenon is most pronounced for samples with a time gap equal to 10 min. In the case of a 0-min time gap, almost no flat pores are seen in the transition zone or through the specimen itself.

## 6. Summary and Conclusions

The influence of various print process parameters on the microstructure of (printed) cementitious materials in fresh and hardened states has been investigated extensively. These results were correlated with mechanical performance derived from the compressive strength and inter-layer bonding strength tests. This study clarified that the surface roughness was significantly affected by increasing the printing speed. Sand particles were forced deeper into the bulk material and created a thicker concentration of paste underneath the top surface of a printed element. This process decreased the roughness and consequently also the mechanical behaviour of the material.

The moisture content of the surface, measured at different inter-layer time intervals, is of major importance and has an influence on the durability and mechanical performance. Focussing on the microstructure, elements fabricated with a 10-min time gap, not only show the relatively highest decrease in moisture content but also show the highest capillary porosity and air void content. The air voids at the layer interface are also more elongated compared to the other series. Furthermore, the number of unhydrated cement particles is the largest in this critical 10-min time interval. The water extracted from the super-positioned layers first serves to rebuild the primary structuration bridges, which are formed during the initial stage of the hydration process.

Most of the findings were in line with the expectations based on the available literature. However, certain process parameters only corresponded quantitatively and during this research it became clear that individual process parameters, for instance, inter-layer interval time, cannot be considered independently of the applied material and other process parameters.

## Figures and Tables

**Table 1:** Composition of 3D printable cementitious material.

Component	CEM I 52.5 N Strong	Sand 0/2	Water	SP
Amount [kg/m^3^]	620.5	1241.0	226.5	0.15 [woc%]

**Table 2:** Chemical and mineralogical composition of cement CEM I 52.5 N [w%].

Composition	CaO	SiO_2_	Al_2_O_3_	Fe_2_O_3_	MgO	Na_2_O	K_2_O	SO_3_
-	64.30	18.30	5.20	4.00	1.40	0.32	0.43	3.50
-	**C_3_S**	**C_2_S**	**C_3_A**	**C_4_AF**	Blaine [m^2^/kg]		Density [kg/m^3^]	
-	71.98	1.75	7.02	12.16	408		3160	

**Table 3:** Average surface roughness R_a_ of elements fabricated with different printing speeds.

Printing Speed [cm/s]	R_a,x_ [-]	Stdev [-]	R_a,y_ [-]	Stdev [-]
1.7	0.95	0.05	0.68	0.15
3.0	0.39	0.07	0.46	0.08

## References

[1] Pegna J. (1997). Exploratory investigation of solid freeform construction. Autom. Constr..

[2] Khoshnevis B. (2004). Automated construction by contour crafting—related robotics and information technologies. Autom. Constr..

[3] Bos F., Wolfs R., Ahmed Z., Salet T. (2016). Additive manufacturing of concrete in construction: Potentials and challenges of 3D concrete printing. Virtual Phys. Prototyp..

[4] Wolfs R.J.M., Bos F.P., Salet T.A.M. (2018). Early age mechanical behaviour of 3D printed concrete: Numerical modelling and experimental testing. Cem. Concr. Res..

[5] Reiter L., Wangler T., Roussel N., Flatt R.J. (2018). The role of early age structural build-up in digital fabrication with concrete. Cem. Concr. Res..

[6] Holt E. (2005). Contribution of mixture design to chemical and autogenous shrinkage of concrete at early ages. Cem. Concr. Res..

[7] Mechtcherine V., Dudziak L. (2012). Effects of superabsorbent polymers on shrinkage of concrete: Plastic, autogenous, drying. Application of Super Absorbent Polymers (SAP) in Concrete Construction: State-of-the-Art Report Prepared by Technical Committee 225-SAP, V. Mechtcherine and H.-W. Reinhardt, Editors.

[8] Boel V. (2006). Microstructuur van zelfverdichtend beton in relatie met gaspermeabiliteit en duurzaamheidsaspecten. Ph.D. Thesis.

[9] De Belie N., Monteny J., Beeldens A., Vincke E., Van Gemert D., Verstraete W. (2004). Experimental research and prediction of the effect of chemical and biogenic sulfuric acid on different types of commercially produced concrete sewer pipes. Cem. Concr. Res..

[10] Khalil N., Aouad G., El Cheikh K., Rémond S. (2017). Use of calcium sulfoaluminate cements for setting control of 3D-printing mortars. Constr. Build. Mater..

[11] Le T.T., Austin S.A., Lim S., Buswell R.A., Gibb A.G.F., Thorpe T. (2012). Mix design and fresh properties for high-performance printing concrete. Mater. Struct..

[12] Kazemian A., Yuan X., Cochran E., Khoshnevis B. (2017). Cementitious materials for construction scale 3D printing: Laboratory testing of fresh printing mixture. Constr. Build. Mater..

[13] Ma G., Wang L. (2018). A critical review of preparation design and workability measurement of concrete material for largescale 3D printing. Front. Struct. Civ. Eng..

[14] Lim S., Buswell R.A., Le T.T., Austin S.A., Gibb A.G.F., Thorpe T. (2012). Developments in construction-scale additive manufacturing processes. Autom. Constr..

[15] Reinhardt H.W., Grosse C.U. (2004). Continuous monitoring of setting and hardening of mortar and concrete. Constr. Build. Mater..

[16] Robeyst N., Grosse C.U., De Belie N. (2009). Measuring the change in ultrasonic p-wave energy transmitted in fresh mortar with additives to monitor the setting. Cem. Concr. Res..

[17] Robeyst N., Gruyaert E., Grosse C.U., De Belie N. (2008). Monitoring the setting of concrete containing blast-furnace slag by measuring the ultrasonic p-wave velocity. Cem. Concr. Res..

[18] Snoeck D. (2015). Self-healing and microstructure of cementitious materials with microfibres and superabsorbent polymers. Ph.D. Thesis.

[19] Perrot A., Rangeard D., Pierre A. (2016). Structural built-up of cement-based materials used for 3D-printing extrusion techniques. Mater. Struct..

[20] Ye G. (2003). Experimental Study and numerical simulation of the development of the microstructure and permeability in cementitious materials. Ph.D. Thesis.

[21] Le T.T., Austin S.A., Lim S., Buswell R.A., Law R., Gibb A.G.F., Thorpe T. (2012). Hardened properties of high-performance printing concrete. Cem. Concr. Res..

[22] Panda B., Chandra Paul S., Jen Tan M. (2017). Anisotropic mechanical performance of 3D printed fiber reinforced sustainable construction material. Mater. Lett..

[23] Audenaert K. (2006). Transportmechanismen in zelfverdichtend beton in relatie met carbonatatie en chloridepenetratie. Ph.D. Thesis.

[24] Masschaele B., Dierick M., Van Loo D., Boone M.N., Brabant L., Pauwels E. (2013). HECTOR: A 240kV micro-CT setup optimized for research. J. Phys. Conf. Ser..

[25] Vlassenbroeck J., Dierick M., Masschaele B., Cnudde V., Van Hoorebeke L., Jacobs P. (2007). Software tools for quantification of X-ray microtomography at the UGCT. Nucl. Instrum. Meth. A.

[26] Marchon D., Kawashima S., Bessaies-Bey H., Mantellato S., Ng S. (2018). Hydration and rheology control of concrete for digital fabrication: Potential admixtures and cement chemistry. Cem. Concr. Res..

[27] Di Carlo T. (2012). Experimental and numerical techniques to characterize structural properties of fresh concrete relevant to contour crafting. Ph.D. Thesis.

[28] Wolfs R.J.M., Bos F.P., Salet T.A.M. (2019). Hardened properties of 3D printed concrete: The influence of process parameters on interlayer adhesion. Cem. Concr. Res..

[29] Van Zijl G.P.A.G., Paul S.C., Tan M.J. Properties of 3D Printable Concrete. Proceedings of the 2nd International Conference on Progress in Additive Manufacturing (Pro-AM 2016).

